# The Chase Metallic Combination Plate

**Published:** 1890-04

**Authors:** 


### The Chase Metallic Combination Plate.

This is a method of constructing an artificial denture with a metallic plate, which shall cover the roof of the mouth and come about to the center of the ridge of the gums. The outer portion and attachment to this plate consists of rubber. The details of manipulation are such that a denture can be easily and in a short time thoroughly constructed. The work from the beginning to the end is so free from dirt and litter which ordinarily pertains to the dental laboratory that it might be done upon a table in the operating room.

After one sees and understands the process, he almost involuntarily says, "Well that is a very simple thing; the wonder is that every one hadn't done it long ago, but then they have not, and, like a great many things, they were simple but useful, but not utilized.

This method of constructing a denture has been closely examined by Dr. L. P. Haskell, of Chicago; than whom there is no better man in the profession to pronounce upon the various kinds of artificial substitutes. The Doctor says "I have given the Chase Combination Plate a careful investigation in all its bearings. I can say while it is not equivalent to a full metal plate, it has most assuredly a great advantage over a rubber plate, and its use will prove beneficial to both patients and dentist, enabling the former to secure a denture far preferable to all rubber plates, and at a much less cost than an all gold plate, and enabling the dentist to receive a better remuneration for his services than when he inserts a rubber plate. The principle is not a new one but the method is, and will readily commend itself to both dentist and patient." When Dr. Haskell pronounces thus confidently upon any material or process, the rest of us will do well to simply acquiesce in his decision.

